# Electrical impedance tomography in congenital heart disease: advancing non-invasive pulmonary perfusion assessment at bedside

**DOI:** 10.1186/s40635-025-00783-3

**Published:** 2025-07-23

**Authors:** Alfio Bronco, Francesco Fazzi, Liliana Amendolagine, Roberta Garberi, Stefano Cattaneo, Floriana Ferrari, Ezio Bonanomi, Giuseppe Foti, Emanuele Rezoagli

**Affiliations:** 1 Pediatric Anesthesiology and Intensive Care Unit, ASST Papa Giovanni XXIII, HPG23 Hospital, Bergamo, Italy; 2 School of Medicine and Surgery, University of Milano-Bicocca, Monza, Italy; 3 Anesthesiology and Intensive Care, Fondazione IRCCS San Gerardo Dei Tintori, Monza, Italy

**Keywords:** Lung perfusion, Congenital heart disease, Electrical impedance tomography, Ventilation, Cardiopulmonary interaction

## Abstract

**Background:**

In congenital heart disease (CHD), the evaluation of pulmonary perfusion remains challenging, particularly in pediatric critically ill patients, where anatomical anomalies significantly impact pulmonary blood flow. We aim at demonstrating the reliability and the accuracy to investigate pulmonary perfusion in the presence of CHD by using electrical impedance tomography (EIT), a non-invasive, bedside, real-time, radiation-free imaging technique that assesses lung ventilation and perfusion.

**Results:**

This methodologies series explores the application of EIT in three pediatric critically ill patients with CHD admitted to the Pediatric Intensive Care Unit at Papa Giovanni XXIII Hospital, Bergamo, Italy: (1) a newborn post-corrective surgery for transposition of the great arteries; (2) an infant post-repair of tetralogy of Fallot with bilateral pulmonary branch stenosis; and (3) an infant with severe hypoxemia following Stage I Norwood–Sano repair. EIT perfusion was performed by injecting a bolus of 0.5 ml/kg of 5% saline through a central venous catheter during an inspiratory hold and was compared to standard imaging techniques that assess pulmonary perfusion. EIT findings were consistent with conventional imaging modalities that are not available at bedside (i.e., computed tomography, magnetic resonance imaging, angiography) or that do not allow regional assessment of lung perfusion and are operator dependent (i.e., ultrasound), demonstrating the reliability and the accuracy of EIT assessment. EIT provided critical insights into ventilation–perfusion dynamics, allowing to identify perfusion defects and guiding clinical decisions.

**Conclusions:**

This clinical investigation highlights the potential of EIT to improve pulmonary perfusion monitoring and clinical management of complex CHD cases in pediatric critically ill patients. Further research is needed to establish standardized protocols and validate the EIT clinical utility in larger cohorts.

**Supplementary Information:**

The online version contains supplementary material available at 10.1186/s40635-025-00783-3.

## Take-home message:

**Table:** 

Electrical impedance tomography (EIT) is a non-invasive, bedside, real-time, radiation-free imaging technique that allows to assess lung perfusion and ventilation/perfusion matching in pediatric patients with congenital heart disease. This may have the potential of monitoring lung perfusion, guide and optimize the clinical management of this specific critically ill patient population.	

## Introduction

In pediatric patients with congenital heart disease (CHD) the estimation of pulmonary perfusion often represents a "black box". Accurate assessment of pulmonary perfusion is crucial as CHD involves both complex intracardiac and extracardiac shunts and sometimes two circulatory systems that are not arranged in series. The altered anatomy of CHD highlights the need to clinically assess and optimize lung–heart interaction, tailor treatment approaches, monitor surgical outcomes, and explore the impact of impaired pulmonary perfusion on prognosis. Various imaging modalities are available to evaluate pulmonary perfusion in CHD patients, including cardiac magnetic resonance imaging (MRI), computed tomography (CT), angiography and quantitative lung perfusion scintigraphy. However, these technologies require logistical organization and are not available at bedside. Echocardiography may overcome these issues, but does not allow to thoroughly assess regional pulmonary perfusion.

Electrical impedance tomography (EIT) is a non-invasive, real-time, radiation-free imaging technique that continuously monitors regional lung ventilation, particularly in critically ill patients, based on electrical bioimpedance variability during a respiratory cycle [1]. Perfusion monitoring can be achieved through EIT waveform analysis in controlled mechanical ventilation after hypertonic saline injection during respiratory hold [2–5]. This method allows conductivity changes due to ventilation to be neglected, while the injected bolus of NaCl solution produces a time–impedance dilution curve that follows typical first-pass kinetics as it passes through the pulmonary circulation. Offline analysis of tracings enables the determination of regional pulmonary ventilation and perfusion distribution, as well as the identification of areas of ventilation–perfusion matching or mismatch (perfused-only or ventilated-only) [3].

Although lung perfusion assessed by EIT has been described in preclinical and clinical reports in adults and in one report involving a child [6], the ventilation–perfusion coupling in children with CHD has not yet been investigated using EIT. In this methodologies series, we describe the application of EIT to assess pulmonary perfusion in three pediatric patients with CHD and confirm our findings by comparing them with standard imaging techniques for lung perfusion detection.

## Materials and methods

EIT was performed using a 16-electrode belt (PulmoVista 500, Dräger, Lübeck, Germany) placed between the 4th and 5th intercostal spaces. All patients were ventilated with lung-protective settings: a positive end-expiratory pressure (PEEP) of 5 cmH₂O, tidal volumes ranging from 6 to 8 mL/kg of predicted body weight, and respiratory rates adjusted according to age to maintain normocapnia. Patients were paralyzed during the EIT analysis, with no spontaneous or assisted respiratory efforts occurring throughout the measurement period. In addition, the mattress was flattened, and pulsation therapy discontinued in order to avoid interference with EIT measurements. Data were acquired at a sampling frequency of 50 Hz and saved for offline analysis. Regional pulmonary perfusion was assessed following the injection of a bolus of 0.5 ml/kg of 5% saline through a central venous catheter during an inspiratory hold and was compared to standard imaging techniques that assess pulmonary perfusion. This approach was chosen to minimize ventilation-related impedance fluctuations and to ensure a stable, recruited lung volume during the bolus transit, consistent with previous contrast-enhanced EIT protocols [3, 7].

Bolus injection of a hypertonic saline solution has been shown to provide improved accuracy and stronger correlation with reference perfusion techniques, such as SPECT, when compared to pulsatility-based perfusion assessments. In particular, Borges et al. [4] demonstrated that bolus tracking offered better agreement in Bland–Altman analysis compared to maps derived from regional pulsatility measurements. Data analysis was conducted with EIT Perfusion Analysis V1.2.0 software (Dräger, Lübeck, Germany). This software employs a blind signal separation algorithm to isolate the physiological components of interest, such as cardiac-related and pulmonary perfusion signals, from background variability. Pixels showing a ventilation or perfusion-related impedance decrease of more than 15% of the maximum within the image were considered ventilated and perfused, respectively. To ensure high-quality data acquisition, the PulmoVista 500 system automatically selects the optimal frequency for current injection within the 80–130 kHz range by identifying the band with the lowest background noise at the time of measurement. Importantly, the EIT data files loaded into the Perfusion software for offline analysis consist of unfiltered raw data, guaranteeing that all offline processing starts from an unaltered and consistent dataset. Supplementary Fig. 1 illustrates a sample impedance curve analyzed with the perfusion software. Beyond this perfusion analysis, we also presented the "time to slope" analysis, which reflects the rate at which saline—and thus blood— accumulates in different lung regions. A higher slope value indicates faster saline accumulation.

We analyzed ventilation and perfusion on a pixel-by-pixel basis to identify regions that were perfused-only, ventilated-only, or matched, the latter defined as pixels exhibiting both ventilation and perfusion signals.

### Case 1—assessment of lung ventilation and perfusion with EIT to evaluate a newborn following corrective surgery for transposition of the great arteries (TGA)

A 12-day-old newborn, weighing 3.4 kg, was diagnosed prenatally with TGA. At birth, the infant required an emergency atrial septostomy to create a bidirectional shunt. The subsequent arterial switch operation was performed at 10 days of life. Following a period of veno-arterial ECMO, the postoperative course was uneventful.

**Fig. 1:**
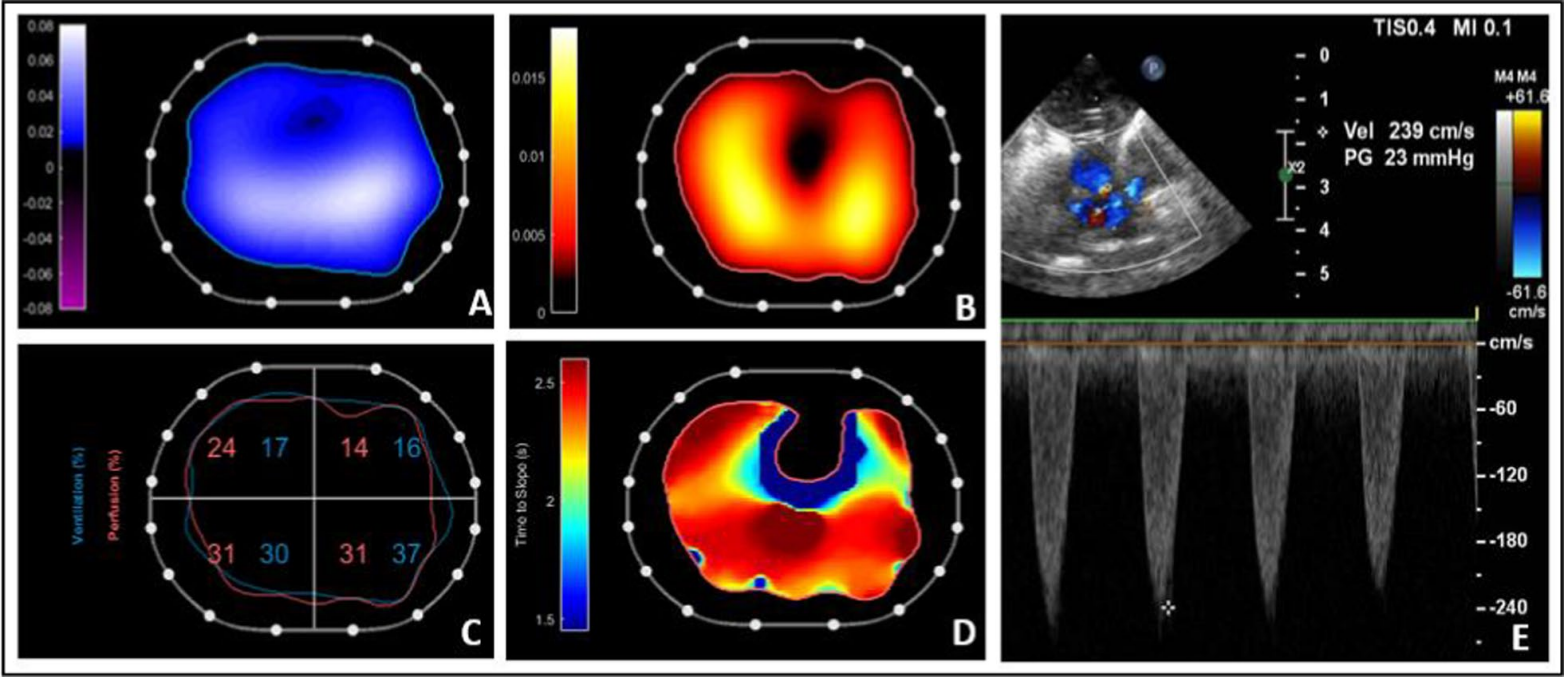
Offline analysis of the time–impedance dilution curve following the injection of a 0.5 ml/kg bolus of 5% saline through a central venous catheter during an inspiratory hold(3): tidal ventilation distribution (**A**) is shown as impedance augmentation where the air enters the lungs (white–blue color scale). Perfusion distribution (**B**) is displayed on a yellow–red color scale, based on impedance reduction as saline bolus passes through the pulmonary circulation. At the bottom left, a summary map (**C**) illustrates the percentage of perfusion (red numbers) and ventilation (blue numbers) across four lung Regions of Interest (ROIs) and allows the identification of regions where ventilation and perfusion are matched (87.2%), regions that are perfused-only (2.5%) and regions that are ventilated-only (10.3%). The fourth map (**D**) shows the different times in which impedance reduces through the thorax during saline distribution: dark blue represents the heart where saline first arrives, followed by yellow and finally red areas of the lungs. The patient was mechanically ventilated with a PEEP of 5 cmH₂O, a tidal volume of 20 mL, and a respiratory rate of 28 breaths per minute. (**E)** Postoperative echocardiography showing a residual gradient across the pulmonary artery branches

Postoperative transthoracic echocardiography identified a low residual gradient across the pulmonary artery branches, likely attributed to the LeCompte maneuver performed during the arterial switch procedure. This maneuver, involving the repositioning of the pulmonary bifurcation anterior to the aorta, may cause structural changes, including shifting the aortic root and altering the position of the pulmonary artery bifurcation. These changes may stretch the vessels, impacting pulmonary flow dynamics. Although the chest X-ray was normal and the residual gradient mild, the patient exhibited suboptimal gas exchange (PaO₂ 80 mmHg on FiO₂ 25%). This clinical discrepancy led to further evaluation with EIT, including assessment of ventilation–perfusion matching. EIT revealed normalized lung perfusion, excellent ventilation–perfusion matching and adequate pulmonary flow, consistent with the echocardiographic findings of a low residual gradient across the pulmonary artery branches. (Fig. 1). A further detailed analysis of sequential perfusion screenshots over time mapping the regional perfusion distribution was reported in Supplementary Fig. 1 and the dynamic sequence of the regional perfusion patterns and temporal distribution within the lungs was provided in Supplementary Video 1.

### Case 2—severe hypoxemia in an infant post-tetralogy of Fallot (TOF) repair with bilateral pulmonary branch stenosis

A newborn weighing 3.5 kg underwent a Blalock–Taussig shunt at 10 days of life due to severe hypoxemia associated with complex TOF, including right pulmonary artery stenosis.

At the age of 7 months and weighing 7.5 kg, the patient had complete surgical repair, including ventricular septal defect closure, transannular patching of the pulmonary valve, and enlargement of the right ventricular outflow tract and right pulmonary artery branch. Postoperatively, the patient showed severe hypoxemia unresponsive to inhaled nitric oxide and invasive respiratory support. Hemodynamic optimization was necessary because of possible residual anatomical abnormalities.

**Fig. 2:**
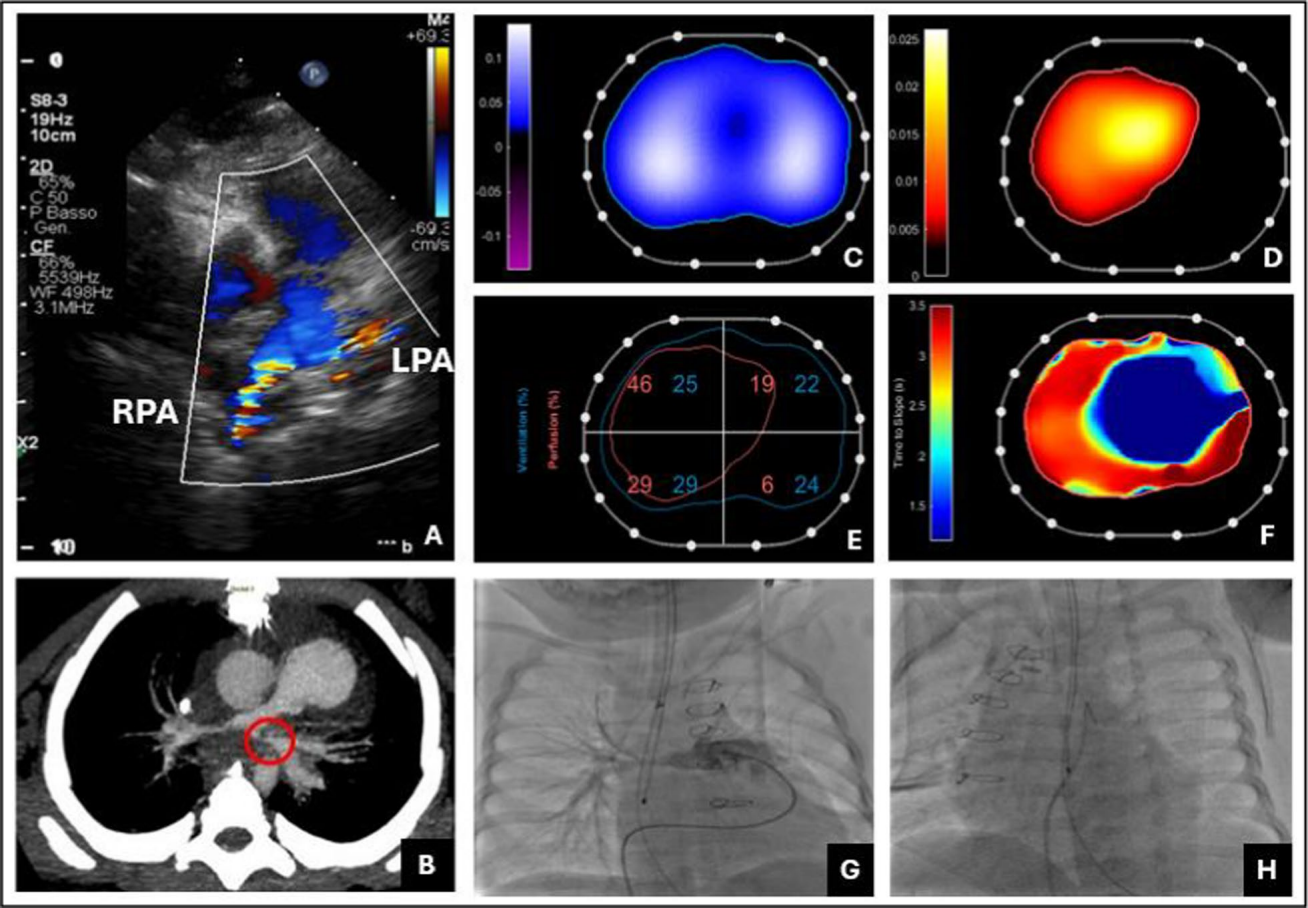
(**A**) Cardiac ultrasound evaluation with evidence of a diminutive right pulmonary artery (RPA) and marked stenosis of the left pulmonary artery (LPA). (**B**) CT scan: RPA stenosis and acute angulation of the LPA. Analysis via EIT of global ventilation (**C**) and perfusion (**D**) distribution. Pixels are classified as perfused if their perfusion-related impedance decreases of more than 15% of the maximum within the image. (**E**) Ventilation and perfusion distribution across every ROI (region of interest) with the corresponding matching of the two components: unmatched ventilation 41.6%, unmatched perfusion 0.9%, ventilation–perfusion matching 57.5%. (**F**) Time to impedance slope in each region: dark blue represents the heart where saline first arrives, followed by yellow and then red areas of the lungs. Cardiac catheterization of the RPA (**G**) and LPA (**H**) at the bottom. The patient was ventilated with a PEEP of 5 cmH₂O, a tidal volume of 40 mL, and a respiratory rate of 25 breaths per minute

Echocardiography showed right ventricular diastolic dysfunction, a right-to-left shunt through a patent foramen ovale, mild pulmonary valve regurgitation, tricuspid insufficiency, and residual right pulmonary artery stenosis, along with a severely angulated left pulmonary artery. CT confirmed these findings, showing severe right pulmonary artery stenosis (2.7 × 2 mm) and acute left pulmonary artery angulation (3 × 2 mm). An EIT ventilation–perfusion study revealed adequate ventilation, adequate right lung perfusion, but highly reduced perfusion in the left lung, providing functional confirmation of the anatomical findings. Angiography was performed to further clarify the underlying anatomical abnormalities. Severe stenosis and acute angulation of the left pulmonary artery was confirmed and deemed unsuitable for intervention. The right pulmonary artery was hypoplastic in its distal portion but, according to EIT, did not significantly affect pulmonary blood flow. It was not treated, and the patient was successfully extubated, maintaining oxygen saturation at a lower acceptable level while achieving stable respiratory function with spontaneous breathing. Future surgical interventions remained under consideration (Fig. 2).

### Case 3—severe hypoxemia in an infant with univentricular heart repair (Stage I Norwood–Sano)

A term newborn, weighing 3.1 kg, was diagnosed prenatally with severe aortic stenosis and left ventricular dysfunction due to myocardial fibroelastosis. On the second day of life, the infant underwent a successful percutaneous valvuloplasty, which reduced the aortic stenosis gradient and stabilized the hemodynamics. However, subsequent cardiac MRI revealed inadequate ventricular function for a biventricular repair. At 14 days of life, the patient underwent a Norwood–Sano procedure, which involved the placement of a Sano conduit between the right ventricle and the main pulmonary artery. Postoperatively, the infant recovered smoothly and was discharged with peripheral oxygen saturation suitable for single-ventricle physiology (SpO₂ 85%).

**Fig. 3:**
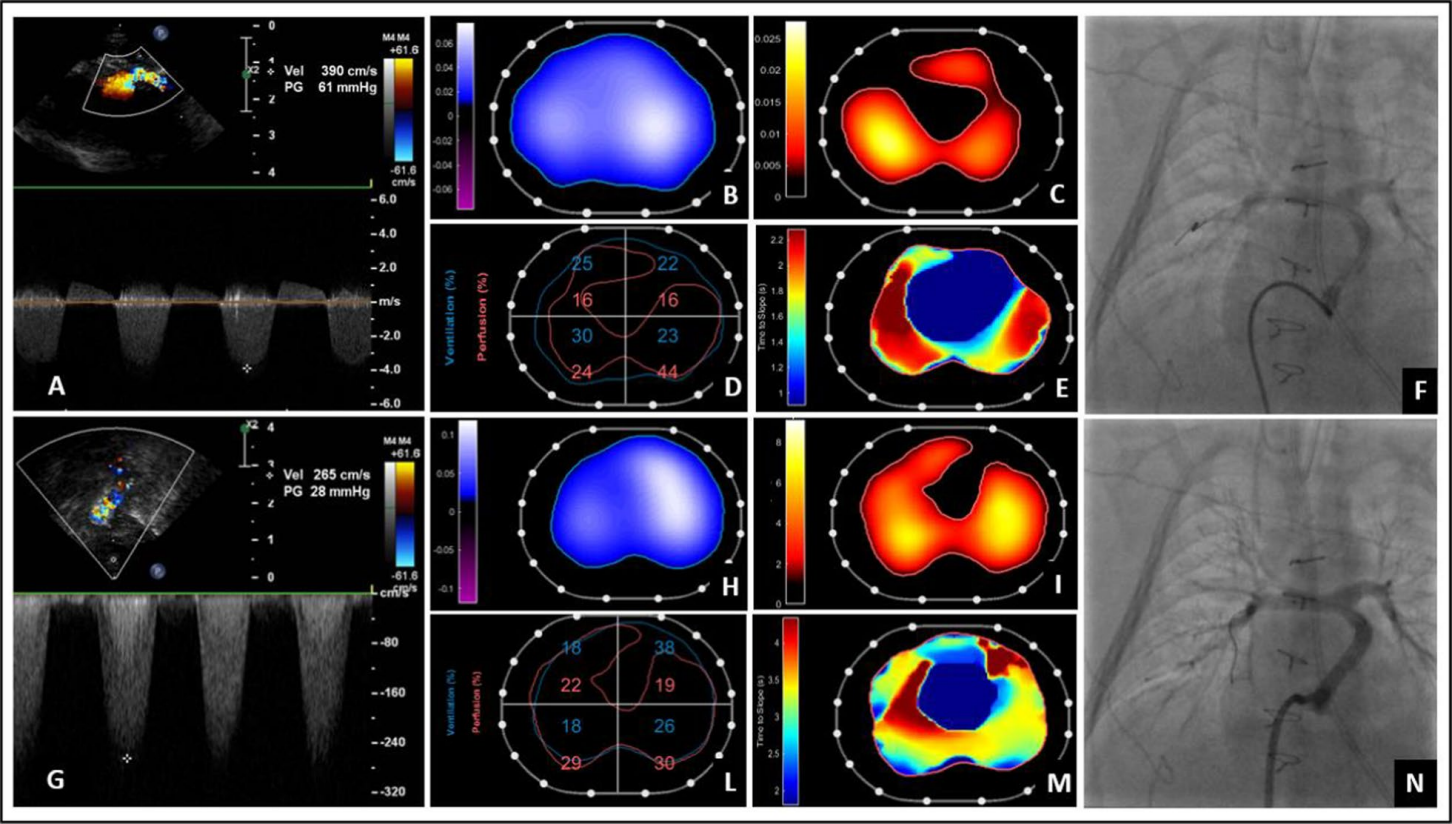
Top: patient evaluation before placement of a drug-eluting stent in the Sano Conduit because of significative stenosis. (**A**) Cardiac ultrasound evaluation showing evidence of a significant maximum gradient on the Sano Conduit (61 mmHg). Analysis via EIT of ventilation (**B**) and perfusion (**C**) global and regional (**D**) distribution confirming the reduced lung perfusion before stent placement, with ventilation–perfusion matching 62.6%, unmatched ventilation 37.2%, unmatched perfusion 0.2%. (**E**) Time (seconds) to impedance slope in each pixel: early bolus arrival is represented by dark blue areas corresponding to the heart region. The subsequent color progression (from green to yellow and red) illustrates the temporal propagation of the bolus through the pulmonary circulation. A darker red area indicates delayed contrast arrival, consistent with impaired flow due to the Sano Conduit stenosis. (**F**) Cardiac catheterization showing stenosis of the Sano Conduit. Bottom: patient evaluation after the placement of a drug-eluting stent in the Sano Conduit. (**G**) Cardiac ultrasound evaluation with a reduced gradient on the Sano Conduit (28 mmHg). Analysis via EIT of ventilation (**H**) and perfusion (**I**) global and regional (**L**) distribution confirming the optimized lung perfusion after stent placement, with increased ventilation–perfusion matching 72.8%, decreased unmatched ventilation 24.7%, and unmatched perfusion 2.5%. (**M**) Time (seconds) to impedance slope showing reduced perfusion delay compared to the pre-treatment evaluation. (**N**) Cardiac catheterization showing complete resolution of the stenosis of the Sano conduit. The patient was ventilated with a PEEP of 5 cmH₂O, a tidal volume of 30 mL, and a respiratory rate of 26 breaths per minute

At the age of 4 months, the patient presented to the Emergency Department with fever and significant desaturation (SpO₂ 50%). Blood tests suggested infection, a chest X-ray showed mild hypotransparency, and echocardiography showed an elevated maximum gradient across the Sano conduit (61 mmHg) and flow acceleration across the neo-aortic arch. Prompt treatment with helmet CPAP (PEEP 5, FiO₂ 80%) provided only a slight improvement in oxygenation (SpO₂ 60–70%). However, hypoxemia persisted despite adequate fluid resuscitation and inotropic support, requiring invasive mechanical ventilation. To further investigate the cause of hypoxemia, a ventilation–perfusion EIT study was performed after intubation and revealed bedside symmetric ventilation across both lungs. However, offline analysis revealed significantly reduced perfusion, indicating a severe perfusion defect rather than a ventilation problem. Additionally, the time-to-peak impedance map demonstrated delayed bolus arrival in the anatomically and functionally compromised segments (Fig. 3E)—findings consistent with a stenosis. A CT angiography study confirmed the presence of stenosis at the level of the Sano conduit, necessitating the placement of a drug-eluting stent. After treatment, oxygenation levels improved, and a subsequent EIT perfusion study demonstrated enhanced bilateral perfusion, optimization of ventilation/perfusion coupling and a reduction in perfusion delay (Fig. 3).

## Discussion

In this methodologies series, we investigated the use of EIT to assess pulmonary perfusion in pediatric patients with congenital heart disease (CHD). Most of the research on EIT has focused on animal models [8–12] and, to our knowledge, there are only a few reports on the application of perfusion EIT in pediatric patients using a filtration technique that separates perfusion and ventilation signals [13, 14]. More recently, a study demonstrated the feasibility of using the saline bolus technique to evaluate ventilation–perfusion matching in a child with acute hypoxic respiratory failure [6]. However, no studies to date have specifically employed EIT with a saline bolus injection for pulmonary perfusion analysis in pediatric patients with CHD.

Based on findings from adult and animal studies [3, 5, 15], we suggest that using a 0.5 mL/kg of 5% saline bolus allows for an accurate assessment of pulmonary perfusion in complex CHD cases. While there is no consensus in the literature on the optimal method for performing saline injections in children, our choice of this specific concentration and volume reflects a careful consideration of both effectiveness and safety. First, previous studies have demonstrated that 5% saline provides excellent signal quality for EIT-based perfusion imaging and this concentration has been widely and effectively used in adult populations [3, 5]. Second, the administered volume (0.5 mL/kg, approximately 0.4 mEq/kg) is substantially lower than clinical doses typically used for therapeutic purposes—amounting to only about 15% of the dose used for intracranial hypertension management (2.7 mEq/kg), which itself causes only a modest rise in serum sodium (+ 3 mEq/L) as reported in a pediatric traumatic brain injury patients [16].

In this methodology investigation, we investigated pulmonary perfusion during an inspiratory hold maneuver. The use of an inspiratory hold during EIT-based perfusion imaging is supported by previous studies, as it reduces ventilation-related impedance noise and provides a stable, recruited lung volume for bolus tracking [3, 7]. While alternative approaches such as end-expiratory [8, 17] or mean airway pressure [15] holds have been proposed, they may carry a higher risk of derecruitment and variable degrees of lung aeration across measurements, especially in injured lungs. Notably, both excessively low and high airway pressures may affect pulmonary vascular resistance and alter regional perfusion. Overdistension may increase resistance by compressing alveolar capillaries, while derecruitment can redirect flow through collapsed units [18]. In this context, ventilation EIT can help identify the optimal lung volume range—minimizing both overdistension and collapse—and thus guide the selection of airway pressures that favor physiologically representative perfusion assessment. Further studies are warranted to compare these strategies directly and support standardization.

In this methodologies series, we demonstrate safety and technical feasibility of this approach, highlighting the novelty and clinical significance of our findings.

The cases presented underscore the potential of EIT as an innovative, non-invasive tool for assessing pulmonary perfusion in pediatric CHD patients. Furthermore, EIT findings were confirmed by standard lung imaging modalities used to assess pulmonary perfusion. EIT offers real-time functional insights at the bedside, which could significantly enhance the management of complex CHD cases. Further research and larger studies are necessary to validate the efficacy of this technique in broader pediatric populations and to explore standardized protocols to perform lung perfusion EIT at the bedside. The use of EIT in pediatric patients with CHD opens a new scenario in the non-invasive lung ventilation/perfusion monitoring at bedside.

## Supplementary Information

Additional file 1.

Additional file 2.

Additional file 3.

## Data Availability

Data are available upon reasonable request to the corresponding author.

## Abbreviations

CHD: Congenital heart disease
CPAP: Continuous positive airway pressure
CT: Computed tomography
ECMO: Extracorporeal membrane oxygenation support
EIT: Electrical impedance tomography
LPA: Left pulmonary artery
MRI: Magnetic resonance imaging
RPA: Right pulmonary artery
TGA: Transposition of great arteries
TOF: Tetralogy of Fallot
VSD: Ventricular septal defect
