# Supplementary material for: Electrical impedance tomography in congenital heart disease: advancing non-invasive pulmonary perfusion assessment at bedside
Source: Intensive Care Med Exp. 2025 Jul 23;13:75. doi: 10.1186/s40635-025-00783-3 (PMC12287481; doi:10.1186/s40635-025-00783-3)
Supplement: Supplementary file 1 — Additional file 1. [file 40635_2025_783_MOESM1_ESM.docx]

**Supplementary material**

**Electrical Impedance Tomography in Congenital Heart Disease: Advancing Non-Invasive Pulmonary Perfusion Assessment at Bedside.**

Alfio Bronco, MD^1^; Francesco Fazzi, MD^1^; Liliana Amendolagine, MD^2^; Roberta Garberi, MD^2^; Stefano Cattaneo, MD^1^; Floriana Ferrari, MD^1^; Ezio Bonanomi, MD^1^; Giuseppe Foti, MD^2,3^, EmanueleRezoagli MD, PhD^2,3^

**Affiliations:**

^1^Pediatric Anesthesiology and Intensive Care Unit, ASST Papa Giovanni XXIII, HPG23 Hospital, Bergamo, Italy

^2^School of Medicine and Surgery, University of Milano-Bicocca, Monza, Italy

^3^Anesthesiology and Intensive Care, Fondazione IRCCS San Gerardo dei Tintori, Monza, Italy

**Corresponding Author**

Emanuele Rezoagli, MD, PhD

School of Medicine and Surgery, University of Milano-Bicocca, Monza, Italy

Department of Emergency and Intensive Care, Fondazione IRCCS San Gerardo dei Tintori, Monza, Italy

Email: [emanuele.rezoagli@unimib.it](mailto:emanuele.rezoagli@unimib.it)

Telephone: +390392339273

**Supplementary Figure 1.** Case 1. Electrical impedance tomography (EIT) perfusion study during an inspiratory pause. The impedance curve is displayed with nine selected sequential time points (0–8), each corresponding to a screenshot of the regional lung perfusion images over time. At baseline (0), no perfusion signal is present. At time point 1, the bolus of hypertonic contrast reaches the cardiac chambers but is excluded from the final perfusion map. From time points 2 to 4, a progressive increase in regional lung perfusion is observed, followed by a gradual decrease in perfusion signal intensity from points 5 to 8, consistent with washout of the contrast agent. In blue, changes in impedance due to ventilation. In red, changes in perfusion due to saline bolus injection.

**Supplementary Video 1.** Case 1. Visualization of the pulmonary perfusion analysis using EIT. The video shows the temporal progression of the bolus as it spreads through the pulmonary vasculature, followed by the gradual washout phase. This dynamic sequence highlights regional perfusion patterns and temporal distribution within the lungs.
