# Supplementary figures and images for: Electrical impedance tomography in congenital heart disease: advancing non-invasive pulmonary perfusion assessment at bedside
Source: Intensive Care Med Exp. 2025 Jul 23;13:75. doi: 10.1186/s40635-025-00783-3 (PMC12287481; doi:10.1186/s40635-025-00783-3)

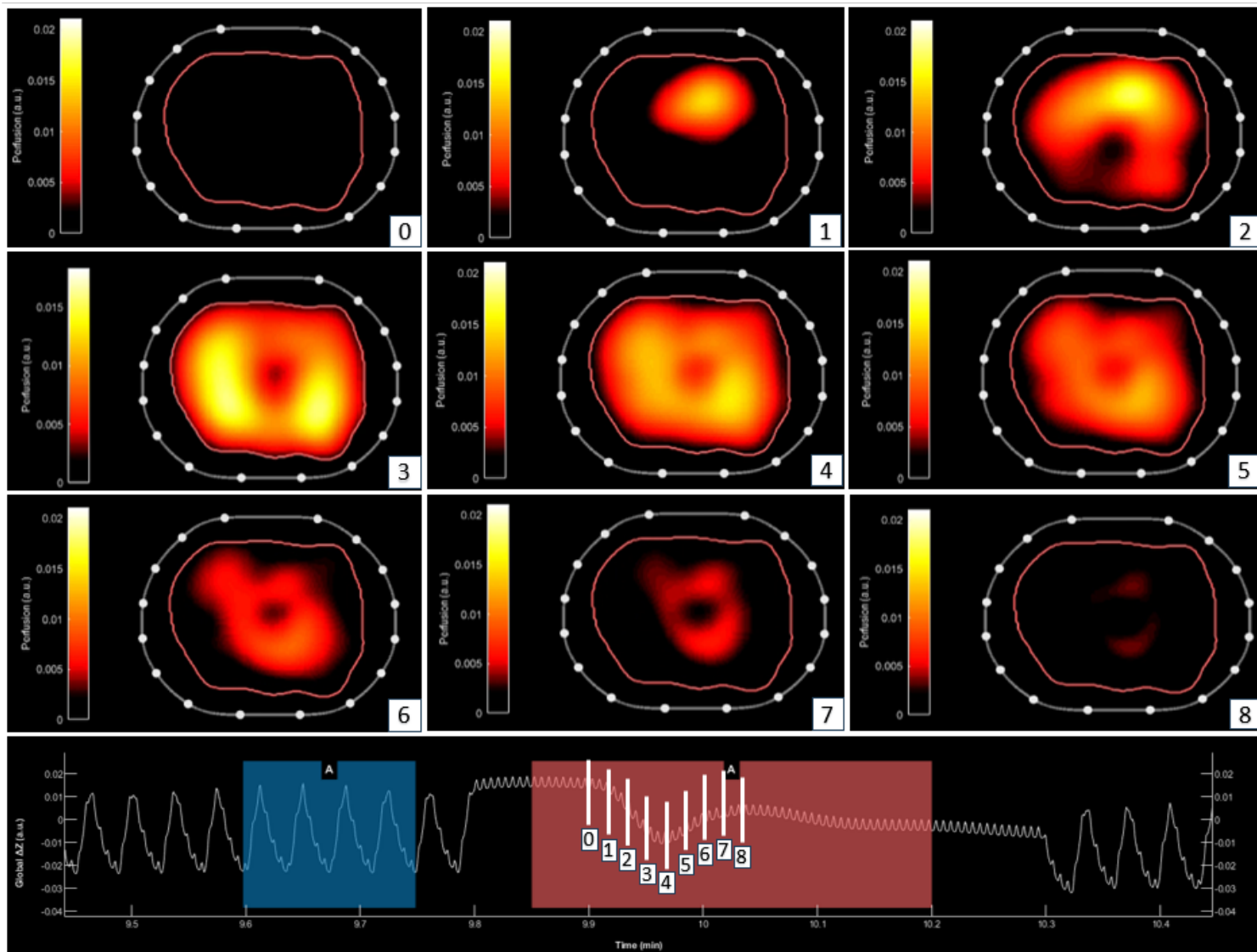

Supplement: Supplementary file 2 — Additional file 2. [file 40635_2025_783_MOESM2_ESM.pdf]
